# Comparative Diagnostic Performance of Ultrasound-Based Risk Stratification Systems in Thyroid Nodule Evaluations by Otolaryngologists

**DOI:** 10.3390/diagnostics16010128

**Published:** 2026-01-01

**Authors:** Jiun-Yi Wu, Ping-Chia Cheng, Ming-Hsun Wen, Chih-Ming Chang, Wu-Chia Lo, Po-Wen Cheng, Po-Hsuan Wu, Li-Jen Liao

**Affiliations:** 1 Department of Otolaryngology Head and Neck Surgery, Far Eastern Memorial Hospital, New Taipei City 220, Taiwan; 2 Department of Biomedical Engineering, National Yang-Ming University, Taipei 112, Taiwan; 3 Head and Neck Cancer Surveillance and Research Study Group, Far Eastern Memorial Hospital, New Taipei City 220, Taiwan; 4 Department of Electrical Engineering, Yuan Ze University, Taoyuan 320, Taiwan

**Keywords:** thyroid nodule, ultrasound, risk stratification system

## Abstract

**Background/Objectives:** Thyroid nodules are a prevalent condition with a high incidence rate of malignancy. Ultrasound (US)-based risk stratification systems have become widely utilized for the evaluation of thyroid nodules, including the American Thyroid Association (ATA) guidelines, the American College of Radiology Thyroid Imaging Reporting and Data System (ACR-TIRADS), the Korean Society of Thyroid Radiology system (K-TIRADS), and the European Thyroid Association system (EU-TIRADS). Our institution has developed a real-time computerized score for evaluating thyroid nodules. This study aims to systematically compare the diagnostic performance of these systems when applied in real time by otolaryngologists, who integrate dynamic US imaging with physical examination. **Methods**: Patients with thyroid nodules who underwent US evaluation, US-guided fine-needle aspiration cytology (FNAC), and subsequent thyroidectomy were included. During each examination, otolaryngologists performed real-time risk categorization according to five US-based systems, with immediate scoring based on dynamic sonographic findings. **Results**: From April 2021 to November 2023, 130 patients were enrolled. For categories 4 and 5, the ATA guidelines had a sensitivity of 96.6% (95% CI: 87.3–100%), specificity of 78.9%, (60.6–97.3%) PPV of 84.6% (70.7–98.5%), NPV of 93.7% (81.9–100%), and accuracy of 88.1% (78.3–97.9%). The sensitivity of the ACR-TIRADS was 95.6% (87.3–100%), the specificity was 78.9% (60.6–97.3%), the PPV was 84.6% (70.7–98.5%), the NPV was 93.7% (81.9–100%), and the accuracy was 88.1% (78.3–97.9%). Both the K-TIRADS and the EU-TIRADS had sensitivities of 95.6% (87.3–100%), specificities of 78.9% (60.6–97.3%), PPVs of 84.6% (70.7–98.5%), NPVs of 93.7% (81.9–100%), and accuracies of 88.1% (78.3–97.9%). The computerized score (>3.3 considered malignant) and TBSRTC (Category 5 or 6) both had sensitivities of 73.9% (56.0–91.9%), specificities of 100%, PPVs of 100%, NPVs of 76.0% (59.3–92.7%), and accuracies of 85.7% (75.1–96.3%). **Conclusions**: Otolaryngologists can achieve highly accurate diagnostic performance when applying standardized ultrasound-based risk stratification systems, and a real-time computerized scoring system provides highly specific supplemental value for immediate clinical decision-making.

## 1. Introduction

Thyroid nodules are common in the general population, with ultrasound (US) detecting nodules in up to 40–50% of individuals [1]. Approximately 5% of these lesions are malignant [2], underscoring the importance of accurate and efficient risk stratification in clinical practice. Because US provides high-resolution, dynamic, and radiation-free visualization of thyroid morphology, it remains the primary imaging modality for initial assessment [3]. The increased accessibility of US has improved detection rates but also raised concerns regarding potential overdiagnosis and unnecessary biopsy of indolent nodules [4,5].

To standardize interpretation and reduce variability, multiple US-based risk stratification systems have been established, including the American Thyroid Association (ATA) guidelines [6], ACR-TIRADS [7], K-TIRADS [8], and EU-TIRADS [9]. These systems share the same objective—distinguishing benign from malignant nodules—but vary in structural design, weighting of sonographic features, and biopsy recommendations. Despite these frameworks, substantial interobserver variability persists, particularly among non-radiologist users [10,11,12], and real-world diagnostic performance is influenced by the examiner’s clinical experience and real-time interpretive ability [13].

In our institution, a real-time computerized scoring system was developed to complement existing guidelines by incorporating four weighted US features—margin, microcalcification, echotexture, and shape—into a single malignancy probability score [14]. This tool is embedded into a PACS-integrated graphical interface that allows for immediate scoring during scanning, offering immediate feedback for clinical decision-making [13,15].

As otolaryngologists increasingly perform office-based ultrasonography for thyroid evaluation, it is crucial to understand how accurately these systems perform when applied by clinicians who integrate both imaging interpretation and physical examination. Unlike radiologists who review static images, otolaryngologists performing real-time scanning can dynamically adjust probe angles, modify compression, correlate US findings with palpable anatomy, and incorporate clinical symptomatology—all factors that may enhance accuracy [16,17,18].

Therefore, the objective of this study was to systematically compare the diagnostic performance of four major international US-based risk stratification systems and our computerized scoring system when applied in real time by otolaryngologists during thyroid ultrasound examinations.

## 2. Materials and Methods

### 2.1. Patients and Methods

**Figure 1:**
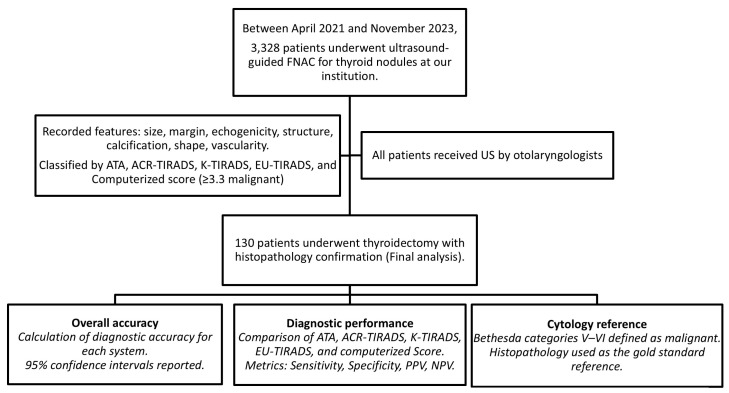
Flow Chart of Patient Enrollment, Ultrasound Evaluation, and Diagnostic Analysis.

This study was approved by the Institutional Review Board of Far Eastern Memorial Hospital (FEMH109074-E). Patients referred to the Department of Otolaryngology–Head and Neck Surgery with suspected thyroid or neck masses were scheduled for ultrasonography in the head and neck US laboratory. When thyroid nodules were detected, patients were invited to participate in this study and provided written informed consent. Clinical data, including age, sex, US findings, FNAC results, and histopathological diagnoses, were collected. This study focused on a surgical cohort; therefore, only patients who subsequently underwent thyroidectomy and obtained a definitive histological diagnosis were included in the final analysis. The flow chart of patient enrollment, ultrasound evaluation, and data analysis is shown in Figure 1.

All US examinations were performed using a high-resolution real-time linear-array transducer with a frequency range of 7–18 MHz (Aplio MX; Toshiba, Tokyo, Japan). The examinations were conducted by two experienced otorhinolaryngologists—head and neck surgeons (abbreviated as L.J.L. and W.C.L.), each of whom had more than 10 years of experience in thyroid imaging and ultrasound elastography [19].The recorded features of each thyroid nodule included the anteroposterior and transverse diameters, margin characteristics (regular or irregular), echogenicity (hyperechoic, isoechoic, or hypoechoic relative to the surrounding thyroid parenchyma), internal structure (solid, predominantly solid, mixed cystic and solid, or cystic, defined as more than 50% cystic component), presence and type of calcification (macrocalcification or microcalcification), and shape (taller-than-wide versus wider-than-tall) [20].

Vascularity patterns were assessed by power Doppler ultrasonography. All the images and reports were stored in the hospital’s PACS (picture archiving and communication system; Marotech, Seoul, South Korea).

To ensure methodological transparency, all interpretations and risk stratification scores were documented in real time during the examination, following a standardized workflow that integrated image acquisition, immediate scoring, FNAC decision-making, and subsequent surgical confirmation. This approach minimized retrospective interpretation bias and ensured that diagnostic data reflected true real-time clinical impressions.

**Figure 2:**
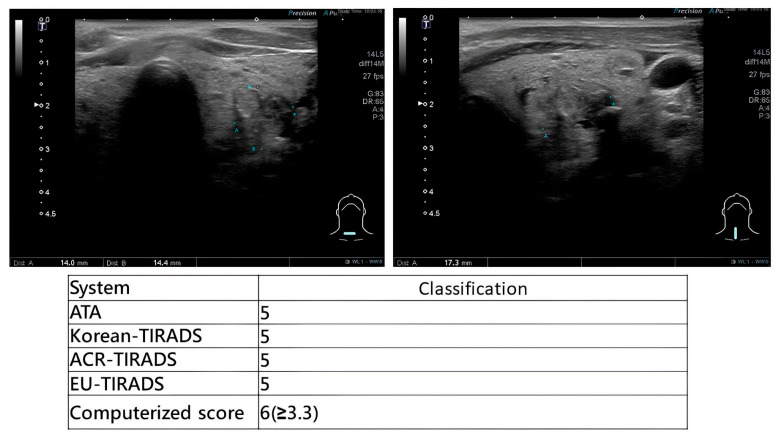
Real-time multi-system risk stratification of a highly malignant thyroid nodule. The computerized score was 6.6, which is categorized as high suspicion.

**Figure 3:**
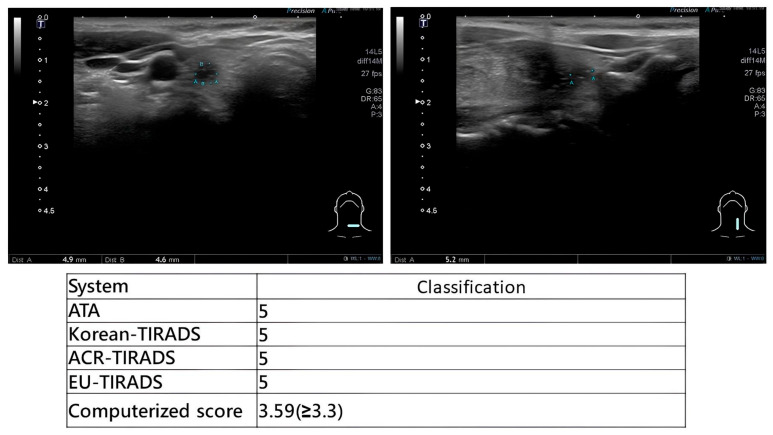
Consistent high-risk stratification of a papillary thyroid carcinoma, strengthened by Computerized scoring (3.59 ≥ 3.3).

**Figure 4:**
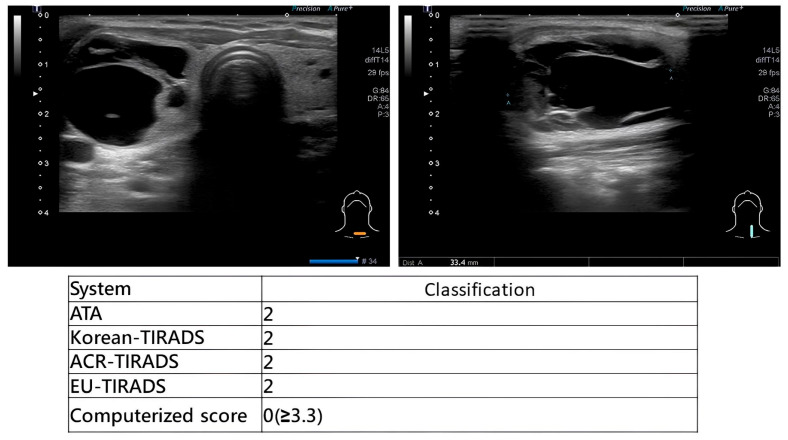
Multi-system risk stratification showing consensus on low suspicion for benign nodule.

During scanning, otolaryngologists applied multiple US-based risk stratification systems concurrently. Representative examples are provided in Figure 2 (high-suspicion papillary thyroid carcinoma), Figure 3 (high-suspicion papillary thyroid carcinoma with computerized scoring = 3.59) and Figure 4 (low-suspicion multinodular goiter). This method ensured uniform application of diagnostic criteria across all evaluated systems.

Each nodule was classified according to five different US-based risk stratification systems:

- The American Thyroid Association (ATA) guidelines [6];
- The American College of Radiology Thyroid Imaging Reporting and Data System (ACR-TIRADS) [7];
- The Korean Thyroid Imaging Reporting and Data System (K-TIRADS) [8];
- The European Thyroid Association TI-RADS (EU-TIRADS) [9];
- The computerized scoring system developed at our institution [14].

For ATA, ACR-TIRADS, K-TIRADS, and EU-TIRADS, categories 4 and 5 were defined as malignant. For the computerized score, a cutoff value of 3.3 or higher was defined as malignant. The computerized score was calculated using the following formula:

**1.25 × margin (0 = regular, 1 = irregular) +

2.03 × microcalcification (0 = absent, 1 = present) +

1.56 × echotexture (0 = mixed cystic–solid, 1 = predominantly solid) +

1.76 × shape (0 = wider-than-tall, 1 = taller-than-wide)**

All patients additionally underwent US-guided FNAC performed by otolaryngologists. Samples were processed using both Liu’s staining and Papanicolaou staining. The cytology results were classified according to the Bethesda System for Reporting Thyroid Cytopathology (TBSRTC), categories I–VI [21]. Bethesda categories V and VI were considered malignant. Final diagnoses were based on histopathology from thyroidectomy specimens, serving as the gold standard for malignancy confirmation.

### 2.2. Statistical Analysis

Statistical analysis was performed using Fisher’s exact test for categorical variables and the Mann–Whitney U test for continuous variables. The diagnostic performance of each system, including the ATA, ACR-TIRADS, K-TIRADS, EU-TIRADS, computerized score, and Bethesda cytology, was assessed by calculating the sensitivity, specificity, positive predictive value (PPV), negative predictive value (NPV), and overall accuracy, with corresponding 95% confidence intervals.

## 3. Results

### 3.1. Main Results

Between April 2021 and November 2023, a total of 3328 patients underwent US-guided FNAC for thyroid nodules at our institution. Among these, 130 patients proceeded to thyroidectomy and were thus eligible for final analysis (Table 1). The mean age was 51.3 years (range: 17–75 years), and the majority of patients were women. Histopathology confirmed 62 malignant nodules (47.69%) and 68 benign nodules (52.31%).

### 3.2. Comparison Between Malignant and Benign Lesions

Analysis of sonographic features demonstrated that irregular margins, heterogeneous internal echotexture, hypoechogenicity, predominantly solid composition, presence of microcalcifications, and avascular or peripheral vascular patterns were all significantly associated with malignancy (*p* < 0.01), consistent with previously reported sonographic predictors of thyroid cancer [22]. Detailed comparisons are summarized in Table 2.

When the four international US-based risk stratification systems (ATA, ACR-TIRADS, K-TIRADS, EU-TIRADS) were evaluated using categories 4–5 as malignant, all demonstrated high and nearly identical diagnostic performance, including:

- Sensitivity: 95.6% (95% CI: 87.3–100%);
- Specificity: 78.9% (95% CI: 60.6–97.3%);
- Positive Predictive Value (PPV): 84.6% (95% CI: 70.7–98.5%);
- Negative Predictive Value (NPV): 93.7% (95% CI: 81.9–100%);
- Accuracy: 88.1% (95% CI: 78.3–97.9%).

These findings are consistent with pooled estimates from recent meta-analyses, which similarly report high sensitivity (85–95%) but only moderate specificity (50–70%) across major TIRADS classifications [23].

Compared with international systems, our computerized scoring system demonstrated a distinctly different diagnostic profile with:

- Sensitivity: 73.9% (95% CI: 56.0–91.9%);
- Specificity: 100%;
- PPV: 100%;
- NPV: 76.0% (95% CI: 59.3–92.7%);
- Accuracy: 85.7% (95% CI: 75.1–93.6%).

Notably, the computerized score achieved the highest specificity and PPV, identical to Bethesda category V/VI cytology (Table 3), suggesting that it functions as a highly specific adjunct to real-time US assessment [24].

This high specificity indicates that the computerized system provides valuable confirmatory information in cases where sonographic suspicion is high, and may be particularly beneficial during immediate physician–patient consultation following FNAC.

## 4. Discussion

This study provides one of the few real-world evaluations of multiple ultrasound (US)-based thyroid nodule risk stratification systems applied directly by otolaryngologists during real-time scanning, rather than by radiologists reviewing static images. Overall, our findings demonstrate that ATA, ACR-TIRADS, K-TIRADS, and EU-TIRADS achieve similarly high sensitivity and good diagnostic accuracy, while the computerized score provides exceptional specificity. Importantly, the present study highlights that otolaryngologists can perform diagnostic thyroid ultrasonography with a level of accuracy comparable to that reported by radiologists and endocrinologists, reinforcing the expanding role of surgeon-performed US in head and neck practice.

### 4.1. Diagnostic Performance of Major International Risk Stratification Systems

The four widely used US-based risk stratification systems—ATA, ACR-TIRADS, K-TIRADS, and EU-TIRADS—share the goal of improving malignancy prediction and standardizing reporting, but they differ substantially in design philosophy, weighting of key sonographic features, and clinical emphasis [23].

The ATA guidelines adopt a pattern-based qualitative approach, emphasizing hypoechogenicity, irregular or microlobulated margins, microcalcifications, and taller-than-wide shape as major suspicious features. This structure is inherently sensitive but often less specific because many benign nodules may mimic these features [25].

In contrast, the ACR-TIRADS employs a quantitative point-based system, assigning weighted scores based on composition, echogenicity, shape, margin, and echogenic foci. This design aims specifically to decrease unnecessary biopsies by incorporating thresholds for FNAC recommendations. Prior studies report that ACR-TIRADS yields fewer FNACs while maintaining reasonable sensitivity [7,26].

K-TIRADS, developed by the Korean Society of Thyroid Radiology, prioritizes echogenicity and solidity, which results in excellent sensitivity in Asian populations with high prevalence of papillary thyroid carcinoma. It also accounts for suspicious lymph nodes, which may increase diagnostic confidence [27].

EU-TIRADS applies a simplified pattern-recognition model, categorizing lesions as high risk (EU-TIRADS 5) when any major suspicious feature is present. This yields a more user-friendly system at the cost of slightly lower specificity [9,28].

Despite these structural differences, our study found that all four systems demonstrated nearly identical diagnostic performance, suggesting that real-time interpretation by a trained examiner may diminish minor system-related variability. In real-time scanning, the examiner can simultaneously cross-reference multiple classification systems, integrate dynamic sonographic tools such as compression, Doppler interrogation, and palpation correlation, and compare suspicious features across frameworks; therefore, a nodule judged malignant in one system is unlikely to be labeled benign in another. This multimodal, synchronous decision-making likely contributes not only to the high concordance observed among the four systems but also to the overall performance of our cohort, in which the sensitivity (95–97%) and specificity (approximately 79%) for ATA, ACR-TIRADS, K-TIRADS, and EU-TIRADS are at the upper end of, or exceed, those reported in large external validation studies. For example, a meta-analysis of 12 studies involving 14,867 nodules demonstrated pooled sensitivities of 0.84 and 0.89 and specificities of only 0.67 and 0.46 for ACR TI-RADS and ATA, respectively [29]. Similarly, a multicenter EU-TIRADS analysis reported a sensitivity of 93.4% but specificity of just 54.6% [30], and another tertiary-center cohort documented ACR-TIRADS sensitivity and specificity of 72% and 68.8% [31]. These comparisons indicate that otolaryngologist-performed, real-time US assessment in our study achieved a more favorable balance between sensitivity and specificity than many contemporary series, likely reflecting the benefits of synchronous feature assessment, cross-system comparison, and dynamic probe optimization during live scanning. However, such repeated cross-system evaluation inevitably increases the operator’s workload, as the examiner is effectively performing multiple parallel classifications during a single examination—an approach that, while improving diagnostic consistency, may be time-consuming and unnecessarily redundant in routine clinical practice [27,32]. To provide clinical clarity, we have summarized the diagnostic elements of each system in Table 4. While the international guidelines (ATA, ACR-TIRADS, K-TIRADS, and EU-TIRADS) provide comprehensive frameworks that often include multiple sonographic patterns, our computerized scoring system employs a more streamlined approach. By utilizing a weighted equation focused on four high-impact features—composition, shape, margin, and microcalcifications—our system prioritizes specificity and objective quantification. Notably, although vascularity was historically considered a predictor of malignancy, it is not included in these major contemporary systems or our own, reflecting a global shift toward more reliable morphologic predictors.

### 4.2. The Unique Value of Otolaryngologist-Performed Real-Time Ultrasonography

A key finding of this study is that otolaryngologists achieved high diagnostic accuracy comparable to radiology-based performance benchmarks, highlighting several advantages unique to clinician-performed real-time ultrasonography. Owing to their detailed understanding of head and neck anatomy, surgeon-operators can identify subtle extrathyroidal extension, correlate palpation findings with imaging, and dynamically adjust probe positioning to better assess margins or microcalcifications [33]. Real-time scanning further allows for adaptive modification in compression, probe angle, and Doppler sensitivity, enhancing visualization of suspicious features that static images may overlook [34].

In addition, clinical context—such as voice changes, neck pressure, swallowing difficulty, or palpable lymphadenopathy—is incorporated immediately at the point of care, improving interpretive accuracy beyond what is possible when imaging is reviewed without direct patient interaction [35]. Continuity of care also contributes meaningfully: when the same clinician performs ultrasonography, FNAC, and surgery, interpretive consistency increases, while interobserver variability decreases [36].

In Taiwan, thyroid ultrasound is partially performed by otolaryngologists because they serve as head and neck surgeons who manage these diseases from diagnosis to operative treatment. The indication for ENT-performed ultrasound is typically a clinical referral for a neck mass, where Point-of-care ultrasound (POCUS) acts as an essential extension of the physical examination [37].

While radiologists also perform real-time scans, the term “dynamic” in this study emphasizes the clinician-led integration of imaging with immediate physical palpation and the patient’s clinical history (such as vocal changes or tactile consistency) at the bedside. This synchronous approach allows for adaptive probe modification based on the surgeon’s clinical suspicion, which may differ from the standardized protocols used in static image review.

Prior studies have confirmed that surgeon-performed ultrasound in head and neck oncology achieves diagnostic accuracy comparable to radiologist interpretation [38]. Collectively, these factors explain why otolaryngologist-performed real-time ultrasonography can yield superior or at least equivalent diagnostic performance relative to radiology workflows that rely on static images and delayed clinical correlation.

### 4.3. Role of the Computerized Real-Time Scoring System

Our institution’s computerized scoring system demonstrated perfect specificity (100%) and PPV (100%), essentially mirroring the performance of Bethesda category V/VI cytology. This is consistent with the design of the computerized score, which incorporates four strongly weighted sonographic predictors of malignancy: irregular margin, microcalcifications, solid echotexture, and taller-than-wide shape.

Because the computerized system is integrated into a PACS-linked graphical interface and generates results instantly during scanning, it provides an immediate confirmatory tool when clinical suspicion is high. This facilitates rapid patient counseling, particularly in cases where FNAC has been performed during the same visit.

While international systems emphasize sensitivity, the computerized system provides specificity-driven supplemental guidance, making it a strong adjunct that reduces unnecessary biopsies and enhances clinical decision-making. However, this specificity-oriented architecture also implies certain limitations. First, because the score relies heavily on classic high-risk features, malignancies that present with less typical sonographic patterns—such as follicular-variant papillary thyroid carcinoma, minimally invasive follicular carcinoma, or medullary carcinoma—may be under-recognized, resulting in lower sensitivity compared with ATA, ACR-TIRADS, K-TIRADS, and EU-TIRADS [27]. Second, the system uses only four dichotomous variables and lacks the multi-tiered, size-adjusted risk stratification present in established frameworks, which limits its ability to provide nuanced malignancy probability estimates or guide FNAC thresholds [9,28]. Third, unlike multi-parameter TIRADS models that consider vascularity, echogenic foci subtypes, spongiform pattern, or lymph node involvement, the computerized score does not incorporate these additional predictors known to enhance the detection of non-classic malignancies [26].

At the same time, the binary, feature-driven structure of our model—classifying each predictor simply as present or absent—offers an important practical advantage. Because the scoring process is objective and does not require complex weighting or multi-tiered pattern recognition, it may serve as a particularly useful introductory tool for trainees or clinicians with limited experience in thyroid ultrasonography. Prior studies have shown that simplified or binary ultrasound decision rules can improve interobserver agreement and support early diagnostic competence among novice operators [13,39].

Taken together, the computerized scoring system functions best as a real-time, high-specificity confirmatory tool, complementing but not replacing the broader sensitivity-oriented international systems. Its performance profile, with high PPV, high specificity, and instantaneous computation, makes it particularly valuable during same-visit FNAC workflows, while its simplicity may additionally support novice operators learning to identify malignant features consistently.

### 4.4. Clinical Implications in the Context of Contemporary Management Trends

The increasing global shift toward active surveillance for low-risk papillary thyroid cancers underscores the need for risk stratification systems that not only detect cancer but also distinguish clinically significant disease. Recent analyses have highlighted that overdiagnosis has become a major public health concern, particularly in regions with widespread access to ultrasonography and rising incidental detection rates [40]. These trends emphasize the importance of stratification frameworks that balance sensitivity with appropriate specificity to avoid unnecessary procedures.

Our findings reinforce that high-sensitivity systems (ATA, K-TIRADS, ACR-TIRADS, EU-TIRADS) are excellent for malignancy triage and initial risk detection, whereas high-specificity tools such as the computerized score may help prevent overtreatment by reducing false-positive classifications that often lead to avoidable FNAC or surgery. This aligns with contemporary management strategies advocating for selective intervention and active surveillance, particularly for small, intrathyroidal, low-risk papillary thyroid carcinomas [41].

The combined use of these systems, interpreted by a skilled clinician, optimizes risk stratification and supports personalized management, whether through FNAC, active surveillance, or surgical planning. This integrated approach is increasingly essential as clinical guidelines evolve toward more nuanced, individualized care pathways for thyroid nodules.

### 4.5. Future Directions: Improving Reproducibility and Integrating Advanced Technologies

As ultrasonography continues to evolve, several emerging technologies may further refine thyroid risk assessment and improve diagnostic reproducibility. Superb microvascular imaging (SMI) offers superior visualization of low-velocity microvascular flow and has demonstrated improved detection of malignant vascular patterns when compared with conventional Doppler technique [42].

However, even with these technological developments, real-time operator expertise, clinical correlation, and physical examination remain irreplaceable. No automated system currently matches the nuanced integration of patient history, palpation findings, dynamic scanning adjustments, and FNAC decision-making that experienced otolaryngologists provide during live evaluation. Thus, while future innovation will undoubtedly enhance diagnostic support, clinician-performed real-time ultrasonography will continue to play a central role in personalized and comprehensive thyroid nodule assessment.

### 4.6. Study Limitations

This study has several limitations. First, the inclusion of only surgically treated patients introduces a significant selection bias, which is reflected in the high malignancy rate (47.69%) of our cohort. This “surgical cohort” design may lead to an overestimation of the specificity and positive predictive value of the computerized scoring system compared to a general screening population. Second, although standardized workflows and uniform scanning protocols were implemented, interobserver variability was not formally quantified, and differences in operator experience may influence generalizability. Third, this study did not evaluate cost-effectiveness or patient-reported outcomes associated with receiving immediate risk stratification feedback during the same clinical encounter, both of which represent important dimensions of real-world clinical utility.

Despite these limitations, this study provides meaningful real-world evidence supporting the diagnostic value of otolaryngologist-performed ultrasonography and highlights the complementary role of computerized real-time scoring in contemporary thyroid nodule assessment.

## 5. Conclusions

Ultrasound-based risk stratification for thyroid nodules achieves optimal diagnostic performance when performed in real time by clinicians who integrate imaging features with physical examination and clinical context. All evaluated systems, including ATA, ACR-TIRADS, K-TIRADS, and EU-TIRADS, demonstrated reliable and highly sensitive diagnostic accuracy when applied by otolaryngologists. These findings underscore that clinician-performed ultrasonography, enhanced by dynamic probe manipulation and immediate clinical correlation, can match the diagnostic precision of radiology-based interpretations. The computerized real-time scoring system further improved specificity and positive predictive value, serving as a valuable confirmatory tool. Integrating international risk stratification systems with the high-specificity computerized scoring system provides a balanced and efficient approach to thyroid nodule assessment. Overall, all the evaluated ultrasound-based risk stratification systems proved reliable for clinical decision-making, particularly when applied by experienced otolaryngologists.

## Figures and Tables

**Table 1:** Clinical characteristics, ultrasound-based risk stratification systems, cytological findings, and final histopathology of the 130 enrolled patients.

Item		N%/Mean ± SD
Sex	Male	32 (25%)
	Female	98 (75%)
Age	Years	51.3 ± 12.5 (17–75)
Side	L	71 (55%)
	R	59 (45%)
Size (short)		1.57 ± 0.79 (0.32–3.52)
Size (long)		2.36 ± 1.46 (0.42–6.45)
ATA	1–3	64 (49%)
	4 or 5	66 (51%)
ACR TIRADS	1–3	64 (49%)
	4 or 5	66 (51%)
K-TIRADS	1–3	63 (48%)
	4 or 5	67 (52%)
EU-TIRADS	1–3	64 (49%)
	4 or 5	66 (51%)
Computerized score (3.3) ^$^	<3.3 ≥3.3	77 (63%) 45 (37%)
Bethesda System	I: Non diagnostic/unsatisfactory	4 (3.08%)
	II: Benign	52 (40%)
	III: Atypia	36 (27.69%)
	IV: Follicular nodule/suspicious follicular nodule	1 (0.77%)
	V: Suspicious for malignancy	25 (19.23%)
	VI: Malignancy	12 (9.23%)
Pathology	Benign	68 (52.31%)
	Malignancy	62 (47.69%)

ATA: The American Thyroid Association (ATA) guidelines [6]; ACR TIRADS: The American College of Radiology Thyroid Imaging Reporting and Data System (ACR-TIRADS) [7]; K-TIRADS: The Korean Thyroid Imaging Reporting and Data System (K-TIRADS) [8]; EU-TIRADS: The European Thyroid Association TI-RADS (EU-TIRADS) [9]; Computerized score: The computerized scoring system developed at our institution [14]. ^$^ Computerized score = 1.25 × margin (regular = 0; irregular = 1) + 2.03 × microcalcification (absent = 0; present = 1) + 1.56 × echo-texture (mixed cystic and solid = 0; predominantly solid = 1) + 1.76 × taller-than-wide shape (absent = 0; present = 1) [14].

**Table 2:** Comparison of ultrasound features, ultrasound-based risk stratification systems, and cytological findings according to benign versus malignant pathology.

		Benign N = 68	Malignancy N = 62	*p* Value
Sex	Male/Female	14 (21%)/54 (79%)	18 (17%)/44 (83%)	0.264
Age		52.6 ± 12.3	49.9 ± 12.8	0.108
Boundary	Clear	56 (88%)	29 (48%)	<0.01
	Vague	8 (12%)	31 (52%)	
Internal echo	Homogenous	42 (66%)	18 (30%)	<0.01
	Heterogeneous	22 (34%)	42 (70%)	
Echogenicity	Hypoechoic	34 (54%)	57 (95%)	<0.01
	Isoechoic	29 (46%)	3 (5%)	
Calcification	No	60 (91%)	27 (45%)	<0.01
	Yes	6 (9%)	33 (55%)	
Architecture	Cystic	20 (30%)	6 (10%)	0.005
	Solid	46 (70%)	54 (90%)	
Hilar echo	Absent	62 (97%)	57 (95%)	0.341
	Oval	2 (3%)	3 (5%)	
Vascular pattern	Avascular	64 (100%)	56 (93%)	0.036
	Spotted	0 (0%)	4 (7%)	
Bethesda system ^@^	I-IV	68 (100%)	25 (40%)	<0.01
	V	0 (0%)	25 (40%)	
	VI	0 (0%)	12 (19%)	
ATA	1–3	35 (51%)	29 (47%)	0.763
	4	21 (31%)	19 (31%)	
	5	12 (18%)	14 (22%)	
ACR TIRADS	1–3	33 (49%)	30 (49%)	0.870
	4	24 (35%)	20 (32%)	
	5	11 (16%)	12 (19%)	
K-TIRADS	1–3	35 (52%)	29 (47%)	0.849
	4	20 (29%)	19 (31%)	
	5	13 (19%)	14 (23%)	
EU-TIRADS	1–3	35 (52%)	29 (47%)	0.765
	4	20 (29%)	18 (29%)	
	5	13 (19%)	15 (24%)	
Computerized score (3.3) ^$^	<3.3	56 (88%)	21 (36%)	<0.01
	≥3.3	8 (12%)	37 (64%)	

^@^ V and VI were characterized as malignancies. ATA: The American Thyroid Association (ATA) guidelines [6]; ACR TIRADS: The American College of Radiology Thyroid Imaging Reporting and Data System (ACR-TIRADS) [7]; K-TIRADS: The Korean Thyroid Imaging Reporting and Data System (K-TIRADS) [8]; EU-TIRADS: The European Thyroid Association TI-RADS (EU-TIRADS) [9];Computerized score: The computerized scoring system developed at our institution [14]. ^$^ Computerized score = 1.25 × margin (regular = 0; irregular = 1) + 2.03 × microcalcification (absent = 0; present = 1) + 1.56 × echo-texture (mixed cystic and solid = 0; predominantly solid = 1) + 1.76 × taller-than-wide shape (absent = 0; present = 1) [14].

**Table 3:** Diagnostic performance of different ultrasound-based risk stratification systems and Bethesda cytology categories in detecting malignant thyroid nodules (Categories 4 and 5 considered malignant).

	Sensitivity (%; 95% CI)	Specificity (%; 95% CI)	Positive Predictive Value (%; 95% CI)	Negative Predictive Value (%; 95% CI)	Overall Accuracy (%; 95% CI)
ATA	95.6 (87.3–100.0)	78.9 (60.6–97.3)	84.6 (70.7–98.5)	93.7 (81.9–100)	88.1 (78.3–97.9)
ACR TIRADS	95.6 (87.3–100.0)	78.9 (60.6–97.3)	84.6 (70.7–98.5)	93.7 (81.9–100)	88.1 (78.3–97.9)
K-TIRADS	95.6 (87.3–100.0)	78.9 (60.6–97.3)	84.6 (70.7–98.5)	93.7 (81.9–100)	88.1 (78.3–97.9)
EU-TIRADS	95.6 (87.3–100.0)	78.9 (60.6–97.3)	84.6 (70.7–98.5)	93.7 (81.9–100)	88.1 (78.3–97.9)
Computerized score (3.3) ^$^	73.9 (56.0–91.9)	100.0 (100.0–100.0)	100.0 (100.0–100.0)	76.0 (59.3–92.7)	85.7 (75.1–96.3)
US-FNA Bethesda (5 or 6) cytology [21]	73.9 (56.0–91.9)	100.0 (100.0–100.0)	100.0 (100.0–100.0)	76.0 (59.3–92.7)	85.7 (75.1–96.3)

ATA: The American Thyroid Association (ATA) guidelines [6]; ACR TIRADS: The American College of Radiology Thyroid Imaging Reporting and Data System (ACR-TIRADS) [7]; K-TIRADS: The Korean Thyroid Imaging Reporting and Data System (K-TIRADS) [8]; EU-TIRADS: The European Thyroid Association TI-RADS (EU-TIRADS) [9]; Computerized score: The computerized scoring system developed at our institution [14]. ^$^ Computerized score = 1.25 × margin (regular = 0; irregular = 1) + 2.03 × microcalcification (absent = 0; present = 1) + 1.56 × echo-texture (mixed cystic and solid = 0; predominantly solid = 1) + 1.76 × taller-than-wide shape (absent = 0; present = 1) [14].

**Table 4:** Comparison of Diagnostic Features and Scoring Methodologies Across Different Systems.

Assessment Item	ATA	ACR-TIRADS	K-TIRADS	EU-TIRADS	Computerized Score
Composition	Yes	Yes	Yes	Yes	Yes
Echogenicity	Yes	Yes	Yes	Yes	No
Shape (Taller-than-wide)	Yes	Yes	Yes	Yes	Yes
Margin	Yes	Yes	Yes	Yes	Yes
Microcalcifications	Yes	Yes	Yes	Yes	Yes
Macrocalcifications	Yes	Yes	Yes	No	No
Vascularity	No	No	No	No	No
Scoring Method *	Pattern	Point-based	Pattern	Pattern	Weighted Equation

Note: * Scoring Method Definitions: Pattern-based (ATA, K-TIRADS, EU-TIRADS): Risk is categorized based on specific combinations of sonographic features. Point-based (ACR-TIRADS): Risk is determined by the total points assigned to individual sonographic features. Weighted Equation (Computerized Score): Risk is calculated as a continuous probability using a statistically weighted logistic regression formula. Regarding calcifications: While major guidelines incorporate various forms of calcifications (e.g., macrocalcifications or rim calcifications), our computerized scoring system specifically focuses on microcalcifications due to their higher positive predictive value for malignancy.

## Data Availability

The original contributions presented in this study are included in this article. Further inquiries can be directed to the corresponding author.

## Abbreviations

The following abbreviations are used in this manuscript:

US	Ultrasound
ATA	American Thyroid Association
ACR-TIRADS	American College of Radiology Thyroid Imaging Reporting and Data System
K-TIRADS	Korean Society of Thyroid Radiology system
EU-TIRADS	European Thyroid Association system
FNAC	Fine-needle aspiration cytology
PPV	Negative predictive value
NPV	Positive predictive value
TBSRTC	The Bethesda System for Reporting Thyroid Cytopathology
GUI	Graphical User Interface
